# Evaluating the risk of ischemic stroke at a young age in patients with autoimmune inflammatory rheumatic diseases: a population-based cohort study in Taiwan

**DOI:** 10.3389/fimmu.2024.1272557

**Published:** 2024-02-09

**Authors:** Ya-Chun Huang, Edward Chia-Cheng Lai, Tzu-Chi Liao, Meng-Yu Weng

**Affiliations:** ^1^ Division of Allergy, Immunology, and Rheumatology, Department of Internal Medicine, National Cheng Kung University Hospital, College of Medicine, National Cheng Kung University, Tainan, Taiwan; ^2^ School of Pharmacy, Institute of Clinical Pharmacy and Pharmaceutical Sciences, College of Medicine, National Cheng Kung University, Tainan, Taiwan

**Keywords:** autoimmune inflammatory rheumatic disease, young stroke, ischemic stroke, incidence, epidemiology

## Abstract

**Background:**

Recent studies have demonstrated an increased incidence of ischemic stroke among patients with certain autoimmune inflammatory rheumatic diseases (AIIRDs). However, the associations between young stroke and AIIRDs have not been fully investigated. This study aimed to evaluate the risk of ischemic stroke among young patients with AIIRDs.

**Methods:**

The National Health Insurance Research Database in Taiwan was utilized to establish cohorts of patients with AIIRDs diagnosed between 2004 and 2015, who were compared with 1,000,000 control participants. Cox proportional hazards regression models were used to calculate the hazard ratio of ischemic stroke and young ischemic stroke for individual AIIRDs after adjustment for relative risk factors.

**Results:**

During the study period, a total of 64,120 patients with AIIRDss and 1,000,000 control patients were identified. The overall mean follow-up time was 5.33 years. There were 223 (0.8%) and 1,923 (0.3%) young ischemic stroke-related hospitalizations among patients with AIIRDs and controls, respectively. The incidence rate of young ischemic stroke was 0.08 in patients with rheumatoid arthritis, 0.08 in patients with Sjögren’s syndrome, 0.26 in patients with systemic lupus erythematosus, 0.17 in patients with idiopathic inflammatory myositis, 0.24 in patients with systemic sclerosis, 0.05 in patients with Behçet’s disease, and 0.44 in patients with systemic vasculitis, versus 0.05 per 100 person-years in the general population. The adjusted hazard ratios for young ischemic stroke were 1.07 (95% CI 0.70–1.43) for rheumatoid arthritis, 1.39 (95% CI 0.94–2.06) for Sjögren’s syndrome, 5.79 (95% CI 4.68–7.17) for systemic lupus erythematosus, 2.07 for idiopathic inflammatory myositis (95% CI 0.98–4.38), 2.79 for systemic sclerosis (95% CI 1.38–5.63), 0.82 for Behçet’s disease (95% CI 0.26–2.55), and 4.15 (95% CI 1.96–8.82) for systemic vasculitis.

**Conclusions:**

Patients younger than 50 years with systemic lupus erythematosus, systemic sclerosis, or systemic vasculitis have a significantly elevated risk of developing ischemic stroke. Further research is needed to elucidate the pathogenesis of accelerated atherosclerosis in these AIIRDs.

## 1 Introduction

Autoimmune inflammatory rheumatic diseases (AIIRDs) include a wide range of related diseases characterized by immune system dysregulation that leads to multiple-system manifestations. In regard to cerebrovascular events, the association between certain AIIRDs and the risk of ischemic stroke has been reported in the cases of systemic lupus erythematosus (SLE) (1), rheumatoid arthritis (RA) (2), systemic sclerosis (SSc) (3), and idiopathic inflammatory myositis (IIM) (4); this cannot be fully explained by traditional cardiovascular risk factors (5, 6). Recent studies have demonstrated an elevated incidence of ischemic stroke among younger people, potentially creating a significant economic burden, as well as causing psychological issues, physical disabilities, and even death (7, 8). This global increase in incidence may result from increased disease awareness, a rising prevalence of cardiovascular risk factors among young adults, and illicit drug use. However, the underlying cause remains undetermined in approximately one-third of young stroke patients, suggesting unknown mechanisms of accelerated atherosclerosis (9).

Due to the nature of chronic inflammation, AIIRDs have been linked with accelerated atherosclerosis and premature cardiovascular disease (10, 11). Previous studies aiming to establish the association between AIIRDs and young stroke have been of limited size, have mostly been confined to specific forms of AIIRD, and have not adjusted for unmeasured confounders such as comorbidities and medication. We hypothesized that young patients with AIIRDs, including RA, SLE, Sjögren’s syndrome (SjS), SSc, Behçet’s disease (BD), IIM, and systemic vasculitis (SV), are at a higher risk of ischemic stroke than the general population.

## 2 Methods

### 2.1 Data source and study population

**Figure 1:**
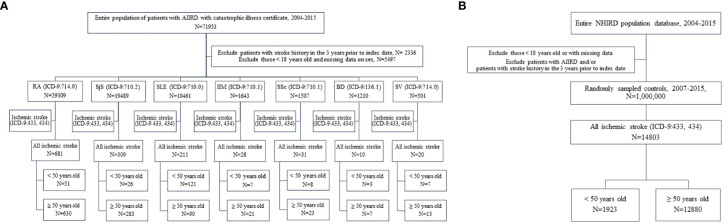
**(A)** Flowchart of patients with autoimmune inflammatory rheumatic diseases (AIIRDs) who developed young ischemic stroke in Taiwan, 2004–2015. **(B)** Flowchart of individuals in the general population who developed young ischemic stroke in Taiwan, 2004–2015. RA, rheumatoid arthritis; SjS, Sjögren’s syndrome; SLE, systemic lupus erythematosus; IIM, idiopathic inflammatory myositis; SSc, systemic sclerosis; BD, Behçet’s disease; SV, systemic vasculitis.

Data were obtained from the National Health Insurance Research Database (NHIRD). Taiwan launched a single-payer mandatory enrollment NHI program in 1995. By 2014, up to 99.99% of Taiwan’s population was participating in this program (12). We established the cohort of AIIRDs patients in our study by using the registry of catastrophic illness patient files in the NHIRD (**Figure 1A**). To avoid financial hardship arising from medical bills, the NHI itemizes 31 groups of catastrophic illnesses, which include most AIIRDs. The attending physician of a patient is obligated to present relevant clinical and laboratory information on application in order to acquire a catastrophic illness certificate (CIC). The application is examined by the review committee based on the classification criteria for individual diseases. Patients are spared from co-payment after CIC approval (13).

The control group consisted of one million individuals in the general population, randomly sampled from the NHIRD from the years 2004 to 2015, as matched control participants (**Figure 1B**). The large-scale cohort and the validation of the catastrophic illness-related diagnosis based on the claims data guarantee that this dataset offers a solid foundation on which to elucidate the incidence of young ischemic stroke among patients with AIIRD. All data in this study were de-identified. This study was approved by the Institutional Review Board of National Cheng-Kung University Hospital, Tainan, Taiwan (A-EX-107-047).

### 2.2 Definitions of AIIRD, young ischemic stroke, traditional risk factors, and related medications

We used the *International Classification of Diseases, Ninth Revision, Clinical Modification* (*ICD-9-CM*) codes to categorize disease diagnoses. The retrospective, population-based cohort of patients with AIIRD in Taiwan included patients with diagnoses of RA (*ICD-9-CM* 714.0), SLE (*ICD-9-CM* 710.0), SjS (*ICD-9-CM* 710.2), SSc (*ICD-9-CM* 710.1), BD (*ICD-9-CM* 136.1), IIM (*ICD-9-CM* 710.3 and 710.4), and SV (*ICD-9-CM* 446). The cohort consisted only of patients aged above 18 years who were approved for the CIC due to their AIIRD between 2004 and 2015; outcomes were compared to those of patients without a diagnosis of AIIRD.

The definition of incident ischemic stroke included cases of occlusion and stenosis of precerebral arteries (*ICD-9-CM* 433) and occlusion of cerebral arteries (*ICD-9-CM* 434), these having been recaptured from the inpatient diagnosis claims data for patients with a principal diagnosis. The diagnostic coding of ischemic stroke in the Taiwan NHIRD has been validated, and the accuracy has been found to be 94% in cases with a principal diagnosis (14). We analyzed each AIIRD cohort as a whole (all ages) and in two separate categories (patients <50 years old and those ≥50 years old). Young ischemic stroke was defined in our study as development of the disease before 50 years of age. Outcomes were compared between patients with and without AIIRD. To avoid selection bias, we included only patients with AIIRD who had no previous admission for ischemic stroke 3 years prior to the follow-up for AIIRD.

The definition of traditional risk factors included diabetes mellitus (*ICD-9-CM 648, 250, 249*), hypertension (*ICD-9-CM 401*), dyslipidemia (*ICD-9-CM 272*), renal failure (*ICD-9-CM 586, 585.9*), and atherosclerosis (*ICD-9-CM 440*). For ischemic stroke-related medications, we used the Anatomical Therapeutic Chemical (ATC) code to identify all relevant medications, including glucocorticoids, antidiabetics, diuretics, beta-blockers, calcium channel blockers, lipid-lowering agents, aspirin, and NSAIDs. Detailed information is provided in the Appendix.

### 2.3 Study design

We established a retrospective, population-based cohort using data from the Taiwan NHIRD in order to identify the incidence of ischemic stroke in patients with AIIRD, as compared to those without, during the period 2004–2015. As matched control participants, the comparison group consisted of 1,000,000 beneficiaries selected randomly from the NHIRD-registered population (2004–2015) after certain exclusions. To minimize selection bias, we applied identical selection criteria and only considered as potential candidates for the control group those for whom complete information on age and sex was available and who were aged ≥18 years between 2004 and 2015. Patients who had AIIRD and/or ischemic stroke within 3 years prior to the index date were also excluded from the control group.

Follow-up started from the date of CIC application for AIIRD, and this date was also matched as the index date for the control group. To recognize the first episode of ischemic stroke (*ICD-9-CM* code 433 or 434), we linked participants to the admission claims data corresponding to the follow-up period. All patients were followed until censoring, including incident ischemic stroke, suspension of coverage, or the end of the study.

### 2.4 Statistical analysis

SAS statistical software (Version 9.4.1; SAS Institute, Cary, NC, USA) was employed for data processing and statistical analysis. We used Cox proportional hazards regression models to evaluate the hazard ratio (HR) for ischemic stroke among individuals with different AIIRDs compared with the control group. We adjusted for all covariates listed in **Table 1**. The Kaplan–Meier method was utilized to compare the cumulative incidence of ischemic stroke in patients with and without AIIRD. A *p*-value of <0.05 was considered significant.

**Table 1:** Baseline demographic characteristics of patients with autoimmune inflammatory rheumatic diseases (AIIRDs) and comparisons.

	RA No. (%)	SjS No. (%)	SLE No. (%)	IIM No. (%)	SSc No. (%)	BD No. (%)	SV No. (%)	Controls No. (%)
**No.**	29,309	19,489	10,461	1,643	1,507	1,210	501	1,000,000
Age (years)								
**Mean**	54.25 (14.00)	54.68 (13.85)	40.48 (15.97)	51.21 (14.46)	52.84 (13.82)	39.23 (12.42)	47.84 (16.72)	43.93 (16.69)
**18*–*34**	2,591 (8.84)	1,644 (8.44)	4,430 (42.35)	214 (13.02)	166 (11.02)	465 (38.43)	125 (24.95)	339,104 (33.91)
**35*–*49**	7,908 (26.98)	4,974 (25.52)	3,302 (31.56)	509 (30.98)	391 (25.95)	490 (40.50)	142 (28.34)	307,071 (30.71)
**50*–*64**	11,824 (40.34)	8,180 (41.97)	1,734 (16.58)	628 (38.22)	645 (42.80)	215 (17.77)	147 (29.34)	228,076 (22.81)
**65*–*79**	5,999 (20.47)	4,036 (20.71)	769 (7.35)	246 (14.97)	280 (18.58)	N/A	74 (14.77)	97,072 (9.71)
***>*80**	987 (3.37)	655 (3.36)	226 (2.16)	46 (2.80)	25 (1.66)	N/A	13 (2.59)	28,677 (2.87)
Sex								
**Female**	22,341 (76.23)	17,545 (90.03)	9,094 (86.93)	1,099 (66.89)	1,079 (71.60)	684 (56.53)	273 (54.49)	502,457 (50.25)
**Male**	6,968 (23.77)	1,944 (9.97)	1,367 (13.07)	544 (33.11)	428 (28.40)	526 (43.47)	228 (45.51)	497,543 (49.75)
Traditional risk factors								
**DM**	3,656 (12.47)	2,167 (11.12)	755 (7.22)	222 (13.51)	183 (12.14)	62 (5.12)	74 (14.77)	72,541 (7.25)
**HTN**	6,965 (23.76)	4,462 (22.89)	1,683 (16.09)	400 (24.35)	376 (24.95)	119 (9.83)	137 (27.35)	123,472 (12.35)
**Dyslipidemia**	5,013 (17.10)	3,783 (19.41)	1,092 (10.44)	303 (18.44)	267 (17.72)	100 (8.26)	78 (15.57)	89,671 (8.97)
**Renal failure**	129 (0.44)	94 (0.48)	137 (1.31)	10 (0.61)	8 (0.53)	4 (0.33)	19 (3.79)	1,588 (0.16)
**Atherosclerosis**	222 (0.76)	138 (0.71)	58 (0.55)	12 (0.73)	38 (2.52)	7 (0.58)	15 (2.99)	2,722 (0.27)
Related medications								
**Glucocorticoids**	23,483 (80.12)	11,382 (58.40)	9,315 (89.05)	1,600 (97.38)	1,091 (72.40)	1,016 (83.97)	443 (88.42)	183,091 (18.31)
**Antidiabetics**	2,863 (9.77)	1,399 (7.18)	771 (7.37)	224 (13.63)	142 (9.42)	39 (3.22)	98 (19.56)	60,892 (6.09)
**Diuretics**	5,802 (19.80)	2,592 (13.30)	3,471 (33.18)	620 (37.74)	491 (32.58)	88 (7.27)	215 (42.91)	54,302 (5.43)
**Beta-blockers**	5,639 (19.24)	4,748 (24.36)	2,294 (21.93)	435 (26.48)	329 (21.83)	192 (15.87)	174 (34.73)	100,846 (10.08)
**CCBs**	6,037 (20.60)	4,183 (21.46)	2,190 (20.93)	443 (26.96)	594 (39.42)	114 (9.42)	192 (38.32)	105,347 (10.53)
**Lipid-lowering agents**	3,103 (10.59)	2,405 (12.34)	942 (9.00)	201 (12.23)	170 (11.28)	57 (4.71)	66 (13.17)	66,288 (6.63)
**Aspirin**	4,564 (15.57)	3,067 (15.74)	2,439 (23.32)	344 (20.94)	485 (32.18)	186 (15.37)	192 (38.32)	71,115 (7.11)
**NSAIDs**	28,844 (98.41)	16,918 (86.81)	9,041 (86.43)	1,446 (88.01)	1,271 (84.34)	1,070 (88.43)	417 (83.23)	614,849 (61.48)

AIIRD, autoimmune inflammatory rheumatic disease; RA, rheumatoid arthritis; SjS, Sjögren’s syndrome; SLE, systemic lupus erythematosus; IIM, idiopathic inflammatory myositis; SSc, systemic sclerosis; BD, Behçet’s disease; SV, systemic vasculitis; DM, diabetes mellitus; HTN, hypertension; CCB, calcium channel blocker; NSAID, non-steroidal anti-inflammatory drug.

N/A (not applicable): There were fewer than three patients with BD in the age groups 65–79 or >80 years old; numbers less than 3 are considered identifiable.

## 3 Results

### 3.1 Baseline characteristics

From 2007 to 2015, a total of 64,120 adult patients with AIIRD and 1,000,000 controls were identified (**Figures 1A, B**). Baseline characteristics including age, sex, traditional cardiovascular risk factors, and ischemic stroke-related medication are given in **Table 1**. There were 29,309 patients with RA; 19,489 with SjS; 10,461 with SLE; 1,643 with IIM; 1,507 with SSc; 1,210 with BD; and 501 with SV identified during the study period (**Figure 1A**). With regard to sex, female patients were greater in number among the AIIRD groups compared with the control group (50.25%), including the groups with RA (76.23%), SjS (90.03%), SLE (86.93%), IIM (66.89%), SSc (71.60%), BD (56.53%), and SV (54.49%). RA, SjS, and SLE were the three most common AIIRDs in Taiwan.

Traditional cardiovascular risk factors for ischemic stroke, such as diabetes mellitus, hypertension, dyslipidemia, renal failure, and atherosclerosis, were more common among patients with RA, SjS, SSc, IIM, and SV than among the general population, which might be attributable to the apparently older mean age of these cohorts. Patients with AIIRD also had a higher frequency of exposure to ischemic stroke-related medications, including glucocorticoids, antidiabetics, diuretics, beta-blockers, calcium channel blockers, lipid-lowering agents, aspirin, and NSAIDs, in comparison with the general population (**Table 1**).

### 3.2 Risk of young ischemic stroke

**Figure 2:**
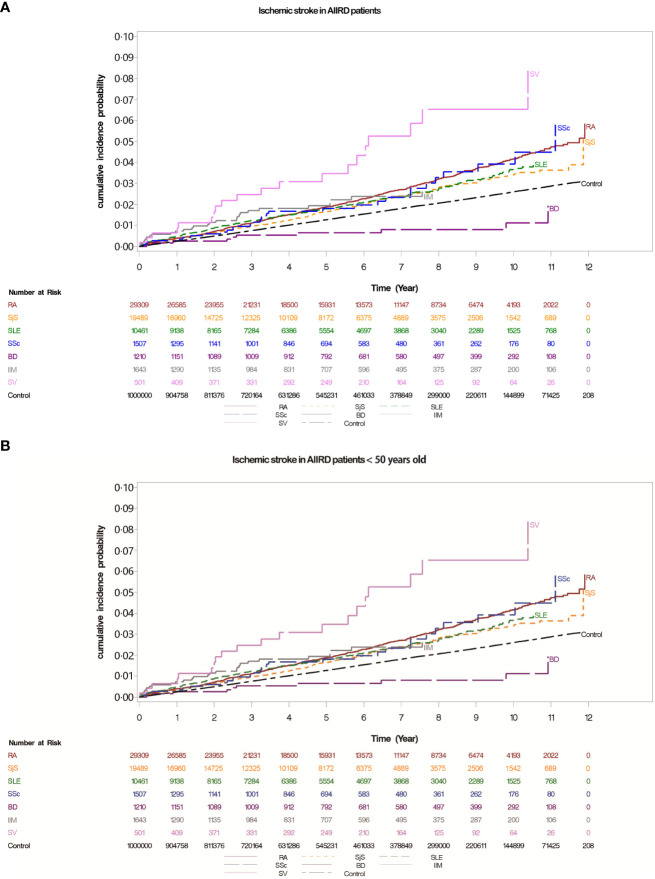
**(A)** Cumulative incidences of ischemic stroke in patients with autoimmune inflammatory rheumatic diseases (AIIRDs), estimated via the Kaplan–Meier method. **(B)** Cumulative incidences of ischemic stroke in patients with autoimmune inflammatory rheumatic diseases (AIIRDs) <50 years old, estimated via the Kaplan–Meier method. RA, rheumatoid arthritis; SjS, Sjögren’s syndrome; SLE, systemic lupus erythematosus; IIM, idiopathic inflammatory myositis; SSc, systemic sclerosis; BD, Behçet’s disease; SV, systemic vasculitis.

The cohort was followed from 1 January 2007 until 31 December 2015; the mean follow-up time was 5.33 years. During this period, there were 223 (0.8%) and 1,923 (0.3%) young ischemic stroke-related hospitalizations among patients with AIIRD and controls, respectively. The incidence rate of young ischemic stroke was 0.08 in patients with RA, 0.08 in patients with SjS, 0.26 in patients with SLE, 0.17 in patients with IIM, 0.24 in patients with SSc, 0.05 in patients with BD, and 0.44 in patients with SV, versus 0.05 per 100 person-years in the general population (**Table 2**, **Figures 2A, B**).

**Table 2:** Evaluation of the risk of young ischemic stroke in patients with autoimmune inflammatory rheumatic diseases (AIIRDs).

	No. of patients	Ischemic stroke events	Total years of follow-up	Stroke incidence rate per 100 PY	Crude HR (95% CI)	*p*-value	Adjusted HR* (95% CI)	*p*-value
**General population**	1,000,000	14,803	5.69	0.26	Reference		Reference	
**RA**	29,309	681	5.70	0.41	1.57 (1.45–1.69)	<0.001	0.99 (0.91–1.07)	0.838
**SjS**	19,489	309	4.68	0.34	1.31 (1.17–1.47)	<0.001	0.90 (0.80–1.00)	0.059
**SLE**	10,461	211	5.52	0.37	1.41 (1.23–1.61)	<0.001	2.10 (1.82–2.41)	<0.001
**IIM**	1,643	28	4.74	0.36	1.39 (0.96–2.01)	0.084	1.14 (0.78–1.65)	0.500
**SSc**	1,507	31	5.08	0.40	1.57 (1.10–2.23)	0.012	1.11 (0.78–1.58)	0.560
**BD**	1,210	10	6.71	0.12	0.47 (0.25–0.88)	0.017	0.94 (0.50–1.75)	0.843
**SV**	501	20	5.13	0.78	2.99 (1.93–4.64)	<0.001	1.88 (1.21–2.92)	0.005
**All AIIRD**	64,120	1,290	5.33	0.38	1.45 (1.37–1.54)	<0.001	1.06 (1.00–1.12)	0.071
Age <50 years								
**General** **population**	646,175	1,923	6.02	0.05	Reference		Reference	
**RA**	10,499	51	6.25	0.08	1.56 (1.18–2.06)	0.0018	1.07 (0.70–1.43)	0.660
**SjS**	6,618	26	5.11	0.08	1.63 (1.11–2.40)	0.014	1.39 (0.94–2.06)	0.104
**SLE**	7,732	121	5.94	0.26	5.32 (4.42–6.39)	<0.001	5.79 (4.68–7.17)	<0.001
**IIM**	723	7	5.68	0.17	3.46 (1.65–7.25)	0.001	2.07 (0.98–4.38)	0.058
**SSc**	557	8	5.97	0.24	4.88 (2.44–9.78)	<0.001	2.79 (1.38–5.63)	0.004
**BD**	955	3	6.88	0.05	0.91 (0.29–2.80)	0.863	0.82 (0.26–2.55)	0.733
**SV**	267	7	5.97	0.44	8.86 (4.22–18.6)	<0.001	4.15 (1.96–8.82)	0.0002
Age ≥50 years								
**General** **population**	353,825	12,880	5.08	0.72	Reference		Reference	
**RA**	18,810	630	5.39	0.62	0.87 (0.80–0.94)	0.0004	0.93 (0.86–1.01)	0.094
**SjS**	12,871	283	4.45	0.49	0.70 (0.62–0.78)	<0.001	0.82 (0.73–0.93)	0.001
**SLE**	2,729	90	4.34	0.76	1.07 (0.87–1.31)	0.53	1.11 (0.90–1.37)	0.323
**IIM**	920	21	4.00	0.57	0.80 (0.52–1.23)	0.308	0.90 (0.59–1.38)	0.632
**SSc**	950	23	4.56	0.53	0.74 (0.49–1.12)	0.16	0.83 (0.55–1.25)	0.368
**BD**	255	7	6.06	0.45	0.63 (0.30–1.32)	0.219	0.97 (0.46–2.04)	0.940
**SV**	501	13	5.13	0.78	2.99 (1.93–4.64)	<0.001	1.88 (1.21–2.92)	0.005
**Age**							1.07 (1.07–1.07)	<0.001
**Male sex**							1.70 (1.64–1.75)	<0.001
**DM**							1.23 (1.15–1.32)	<0.001
**HTN**							1.24 (1.19–1.29)	<0.0001
**Dyslipidemia**							0.89 (0.85–0.93)	<0.0001
**Renal failure**							1.51 (1.28-1.78)	<0.0001
**Atherosclerosis**							1.08 (0.94–1.23)	0.28
Related medication								
**Glucocorticoids**							1.07 (1.03–1.11)	<0.001
**Antidiabetics**							1.49 (1.39–1.60)	<0.001
**Diuretics**							1·20 (1.15–1.24)	<0.001
**Beta-blockers**							1.20 (1.16–1.25)	<0.001
**CCBs**							1.26 (1.21–1.31)	<0.001
**Lipid-lowering agents**							1.11 (1.05–1.17)	<0.001
**Aspirin**							1.34 (1.29–1.39)	<0.001
**NSAIDs**							0.98 (0.95–1.02)	0.33

AIIRD, autoimmune inflammatory rheumatic disease; RA, rheumatoid arthritis; SjS, Sjögren’s syndrome; SLE, systemic lupus erythematosus; IIM, idiopathic inflammatory myositis; SSc, systemic sclerosis; BD, Behçet’s disease; SV, systemic vasculitis; DM, diabetes mellitus; HTN, hypertension; CCB, calcium channel blocker; NSAID, non-steroidal anti-inflammatory drug; CI, confidence interval; HR, hazard ratio.

*Adjusted for age, sex, DM, HTN, dyslipidemia, renal failure, atherosclerosis, and use of glucocorticoids, antidiabetics, diuretics, beta-blockers, calcium channel blockers, lipid-lowering agents, aspirin, and non-steroidal anti-inflammatory drug.

**Table 2** shows that patients with AIIRD had an overall higher risk of developing ischemic stroke compared with the general population (crude HR: 1.45, 95% CI 1.37 to 1.54); the adjusted HR was slightly lower but remained significant after adjustment for age, sex, traditional cardiovascular risk factors for ischemic stroke, and use of relevant medication (adjusted HR: 1.06, 95% CI 1.0 to 1.12). With respect to individual forms of AIIRD, the crude HRs for overall risk of hospitalization for ischemic stroke compared to the control group were significant in patients with RA (1.57, 95% CI 1.45 to 1.69), SjS (1.31, 95% CI 1.17 to 1.47), SLE (1.41, 95% CI 1.23 to 1.61), SSc (1.57, 95% CI 1.10 to 2.23), and SV (2.99, 95% CI 1.93 to 4.64). After adjustment, the overall risk of hospitalization for ischemic stroke was elevated only in patients with SLE (2.10, 95% CI 1.82 to 2.41) and SV (1.88, 95% CI 1.21 to 2.92) compared with their control group. We also identified the risk factors for ischemic stroke-related hospitalization in patients with AIIRD, which include increased age, male sex, DM, HTN, renal failure, and use of related medication (**Table 2**).

Furthermore, we divided the AIIRD cohort and control group by age (younger vs. older than 50 years). For all individual AIIRDs, incidence rates of hospitalization for ischemic stroke were higher in patients ≥50 years old than in patients <50 years old. The crude HRs for ischemic stroke were significant among young patients with RA (1.56, 95% CI 1.18 to 2.06), SjS (1.63, 95% CI 1.11 to 2.40), SLE (5.32, 95% CI 4.42 to 6.39), IIM (3.46, 95% CI 1.65 to 7.25), SSc (4.88, 95% CI 2.44 to 9.78), and SV (8.86, 95% CI 4.22 to 18.6) compared with controls of the same age; however, in patients aged ≥50 years, elevated risk of ischemic stroke compared to the controls was only observed in those with SV (crude HR: 2.99, 95% CI 1.93 to 4.64). After adjustment for traditional cardiovascular risk factors and use of ischemic stroke-related medications listed in **Table 1**, the HRs for hospitalized ischemic stroke indicated that the risk was significantly higher compared to the controls among patients <50 years old with SLE (5.79, 95% CI 4.68 to 7.17), SSc (2.79, 95% CI 1.38 to 5.63), and SV (4.15, 95% CI 1.96 to 8.82). Among patients ≥50 years old, the risk of incident ischemic stroke was only significantly higher compared to the general population in those with SV (1.88, 95% CI 1.21 to 2.92) (**Table 2**, **Supplementary Figure 1**).

## 4 Discussion

AIIRDs are a group of disorders characterized by chronic inflammation and vasculopathy. Chronic inflammation and vasculopathy can cause increased atherosclerosis and thrombosis formation. Stroke is the leading cause of death and long-term disability worldwide (15). Since stroke is often disabling, young stroke poses an enormous threat to socioeconomic stability. Although there have been studies comparing young stroke in patients with AIIRD and individuals without such disorders, no explicit definition of young ischemic stroke has been established. However, we defined “young stroke” as stroke in patients aged 18–49 years, this definition having been used widely in previous studies (8, 16, 17). Our study elucidated the risk of ischemic stroke-related hospitalization among young patients with AIIRD. We identified approximately a six-fold increase in the risk of incident young stroke for SLE, a three-fold increase for SSc, and approximately a four-fold rise for SV relative to the general population. The results indicated an age-dependent pattern of ischemic stroke risk, in which the adjusted hazard ratio (aHR) was significantly higher for AIIRD patients under 50 years old, while a significant difference was not observed for AIIRD patients aged 50 years or older.

The current study illustrates the strong link between SLE and young ischemic stroke, which echoes previous findings (16). Mechanisms of accelerated atherosclerosis in patients with SLE may include immune-mediated endothelial damage with vascular injury, antiphospholipid antibody-induced thrombosis, and corticosteroid therapy. Two meta-analyses have reached the same conclusion that the risk of any stroke is higher in patients with SLE aged under 50 years (17, 18). However, the abovementioned studies combined different stroke types into composite outcomes. Through the use of a strict definition and methodology, our study emphasizes that young SLE patients are associated with a nearly six-fold increase in risk of developing ischemic stroke.

Systemic sclerosis is characterized by vasculopathy, which features endothelial cell dysfunction, vascular remodeling, and vessel wall fibrosis; vasculopathy is a condition that accelerates atherosclerosis and leads to early cardiovascular disease. Published population-based studies have recognized SSc as a risk factor for multiple cardiovascular diseases (3, 19), yet these studies provided limited information on ischemic stroke risk for young adult patients. To our knowledge, ours is the first study to determine that young patients with SSc are at an approximately three-fold increased risk of ischemic stroke compared with the general population.

A strong association between SV and young ischemic stroke was observed in the current study. The pathogenesis of accelerated atherosclerosis in patients with SV may result from endothelial cell activation triggered by certain autoantigens (20) and specific autoantibodies (21, 22), thereby promoting atherosclerosis. Existing studies have established the association between giant cell arteritis and different subtypes of stroke (23, 24). Previous population-based studies have also aimed to clarify the association between ANCA-associated vasculitis and ischemic stroke, with inconsistent results. Avina-Zubieta et al. observed a non-statistically significant trend toward elevated risk of ischemic stroke in patients with granulomatous polyangiitis (GPA) (25), while Mourguet et al. reported an elevated incidence of ischemic stroke in patients with GPA and microscopic polyangiitis (26). Studies focusing on young ischemic stroke in patients with SV have been scarce. Our findings suggested that systemic vasculitis is linked to a significant (four-fold) increase in risk of ischemic stroke among young patients and a significant (two-fold) increase in risk among patients aged more than 50 years.

Population-based studies with large sample sizes have indicated that rheumatoid arthritis is a risk factor for ischemic stroke. Solomon et al. observed a near-doubling of the event rate of ischemic stroke in patients with RA (2). Holmqvist et al., using the same definition of stroke, observed a detectable increase in risk of incident ischemic stroke only after more than 10 years since RA diagnosis (27). Lindhardsen et al., utilizing the Danish civil registration system, reported an elevated risk of stroke in patients with RA based on pooling of hemorrhagic and ischemic stroke together into composite results (28). After adjustment for traditional cardiovascular risk factors and use of related medications, our study results indicated that RA does not increase the risk of ischemic stroke, as we observed a similar risk level in the general population. Aside from racial and ethnic disparities among patients with RA (29), variability between studies may be a possible explanation for this discrepancy in findings.

The current study has revealed that SjS is not associated with young ischemic stroke, which echoes the results of previous studies. Kang and Lin and Chiang et al. have reported that patients with SjS do not suffer from a higher risk of CVA compared with the general population (30, 31). However, we discovered that patients with SjS ≥50 years old had a decreased risk of ischemic stroke in comparison with their control group. Our result echoes that of a previous study in which Chiang et al. reported that the hazard ratio for ischemic stroke in SjS patients aged ≥60 years was 0.74 (95% CI 0.52–1.05, *p* = 0.09) (31). Further investigation is needed to elucidate the possible mechanism.

In contrast, the adjusted HR of dyslipidemia is negatively correlated with ischemic stroke in our study. The result may only indicate an association rather than a causal relationship. Possible explanations for our findings include the use of different operational definitions and the lipid paradox, i.e., an inverse relationship between lipid levels and cardiovascular risk in the presence of active inflammation.

According to our research, young adults with IIM do not have a higher risk of ischemic stroke, which is consistent with published research (4). In contrast, Moshtaghi-Svensson et al. observed the highest risk of stroke in younger patients with IIM, which might be attributable to unmeasured confounders such as medications (32). Our study results suggested that BD is not correlated with ischemic stroke, and that this is the case not only in younger but also in older cohorts. A nationwide population-based study in Taiwan found that patients with BD had a 2.77-fold greater risk of ischemic stroke compared with a control group. These inconsistent findings could be explained by the fact that the study combined several types of cerebrovascular disease to form composite outcomes (33).

First among the strengths of our study is the large-scale population cohort. The NHIRD is a sizeable database providing sufficient statistical power for the study of rare conditions such as AIIRD. Second, we adjusted for confounders such as traditional cardiovascular risk factors and use of related medication. Third, the verified CIC-related diagnoses in the claims data ensured that the incidence of young ischemic stroke among patients with individual AIIRDs could be estimated with high accuracy. Fourth, we estimated the incidence of ischemic stroke only in patients with AIIRD who had no previous admission for ischemic stroke during the 3 years prior to follow-up for AIIRD, thus preventing potential risk overestimation due to selection bias. In conclusion, we have provided robust evidence of the association between young ischemic stroke and AIIRD via this population-based study, with adequate follow-up length and strict methodology.

There are several limitations to this study. No laboratory data were available as part of the claims data to which our study referred, preventing us from analyzing in detail the predictive factors for young ischemic stroke. We included only patients with a CIC for AIIRD; as a result, any patient with AIIRD who had not applied for an associated CIC would not have entered the cohort. Nevertheless, patients would have been spared from co-payment after CIC approval; therefore, the number of patients with AIIRD but no CIC was probably low. In addition, despite our adjustment for use of related medications at baseline, dosage-related or treatment duration-related effects of medications on stroke could not be evaluated. Although we adjusted for use of aspirin, other antiplatelets and anticoagulants may also have a confounding effect. Furthermore, we did not adjust for previous coronary artery disease as a risk factor, which may also have had an effect on our study results. However, since we excluded patients with ischemic stroke during the 3 years prior to follow-up in both the AIIRD cohort and controls, the effect is likely to be limited. Due to the use of the NHIRD, information on variables such as body mass index, exercise and physical activity, tobacco use, diet, and disease severity was unavailable; all of these factors may also have an impact on the incidence of ischemic stroke. Since the underlying mechanisms of young ischemic stroke among patients with AIIRD are complex and multifactorial, there may still be unmeasured confounders in spite of the comprehensive set of adjustments made.

## 5 Conclusions

Patients with SLE, SSc, and SV have an elevated risk of developing ischemic stroke at a young age. Our finding underlines the need to provide monitoring for ischemic stroke in these patients as part of clinical practice. Future research is required to elucidate the pathogenesis of the increased risk of young ischemic stroke in patients with these diseases.

## Data availability statement

The dataset supporting the conclusions of this article is(are) available in the the National Health Insurance Research Database (NHIRD) published by Taiwan National Health Insurance (NHI) Bureau. Due to legal restrictions imposed by the government of Taiwan in relation to the “Personal Information Protection Act”, data cannot be made publicly available. Requests for data can be sent as a formal proposal to the NHIRD (http://nhird.nhri.org.tw).

## Ethics statement

This study was approved by the Institutional Review Board of National Cheng-Kung University Hospital, Tainan, Taiwan (A-EX-107-047). The studies were conducted in accordance with the local legislation and institutional requirements. Written informed consent for participation was not required from the participants or the participants’ legal guardians/next of kin in accordance with the national legislation and institutional requirements.

## Author contributions

YH: Investigation, Writing – original draft. EL: Conceptualization, Data curation, Formal Analysis, Methodology, Writing – review & editing. TL: Data curation, Methodology, Writing – review & editing. MW: Conceptualization, Data curation, Funding acquisition, Investigation, Resources, Validation, Writing – review & editing.

## Supplementary material

Supplementary Figure 1 Cumulative incidences of ischemic stroke in patients with autoimmune inflammatory rheumatic diseases (AIIRDs) ≥50 years old, estimated via the Kaplan–Meier method. RA, rheumatoid arthritis; SjS, Sjögren’s syndrome; SLE, systemic lupus erythematosus; IIM, idiopathic inflammatory myositis; SSc, systemic sclerosis; BD, Behçet’s disease; SV, systemic vasculitis.

## Appendix: List of abbreviations, ICD-9 Diagnosis Codes and ATC codes

AIIRD, autoimmune inflammatory rheumatic disease; RA, rheumatoid arthritis; SjS, Sjögren’s syndrome; SLE, systemic lupus erythematosus; IIM, idiopathic inflammatory myositis; SSc, systemic sclerosis; BD, Behçet’s disease; SV, systemic vasculitis; DM, diabetes mellitus; HTN, hypertension; CCB, calcium channel blocker; NSAID, non-steroidal anti-inflammatory drug; CI, confidence interval; HR, hazard ratio; ICD-9, International Classification of Diseases: Ninth Revision; NHIRD, National Health Insurance Research Database.

ICD diagnosis codes: RA (*ICD-9-CM* 714.0), SLE (*ICD-9-CM* 710.0), SjS (*ICD-9-CM* 710.2), SSc (*ICD-9-CM* 710.1), BD (*ICD-9-CM* 136.1), IIM (*ICD-9-CM* 710.3 and 710.4), SV (*ICD-9-CM* 446), diabetes mellitus (*ICD-9-CM 648, 250, 249*), hypertension (*ICD-9-CM 401*), dyslipidemia (*ICD-9-CM 272*), renal failure (*ICD-9-CM 586, 585.9*), and atherosclerosis (*ICD-9-CM 440*).

ATC (Anatomical Therapeutic Chemical) codes: glucocorticoid (H02), anti-diabetic (A10), diuretic (C03), beta-blocker (C07), calcium channel blocker (C08), lipid-lowering agent (C10), aspirin (B01AC06, N02BA01, N02BA51, N02BA71), and NSAID (M01A).
