# Supplementary figures and images for: Evaluating the risk of ischemic stroke at a young age in patients with autoimmune inflammatory rheumatic diseases: a population-based cohort study in Taiwan
Source: Front Immunol. 2024 Feb 9;15:1272557. doi: 10.3389/fimmu.2024.1272557 (PMC10884215; doi:10.3389/fimmu.2024.1272557)

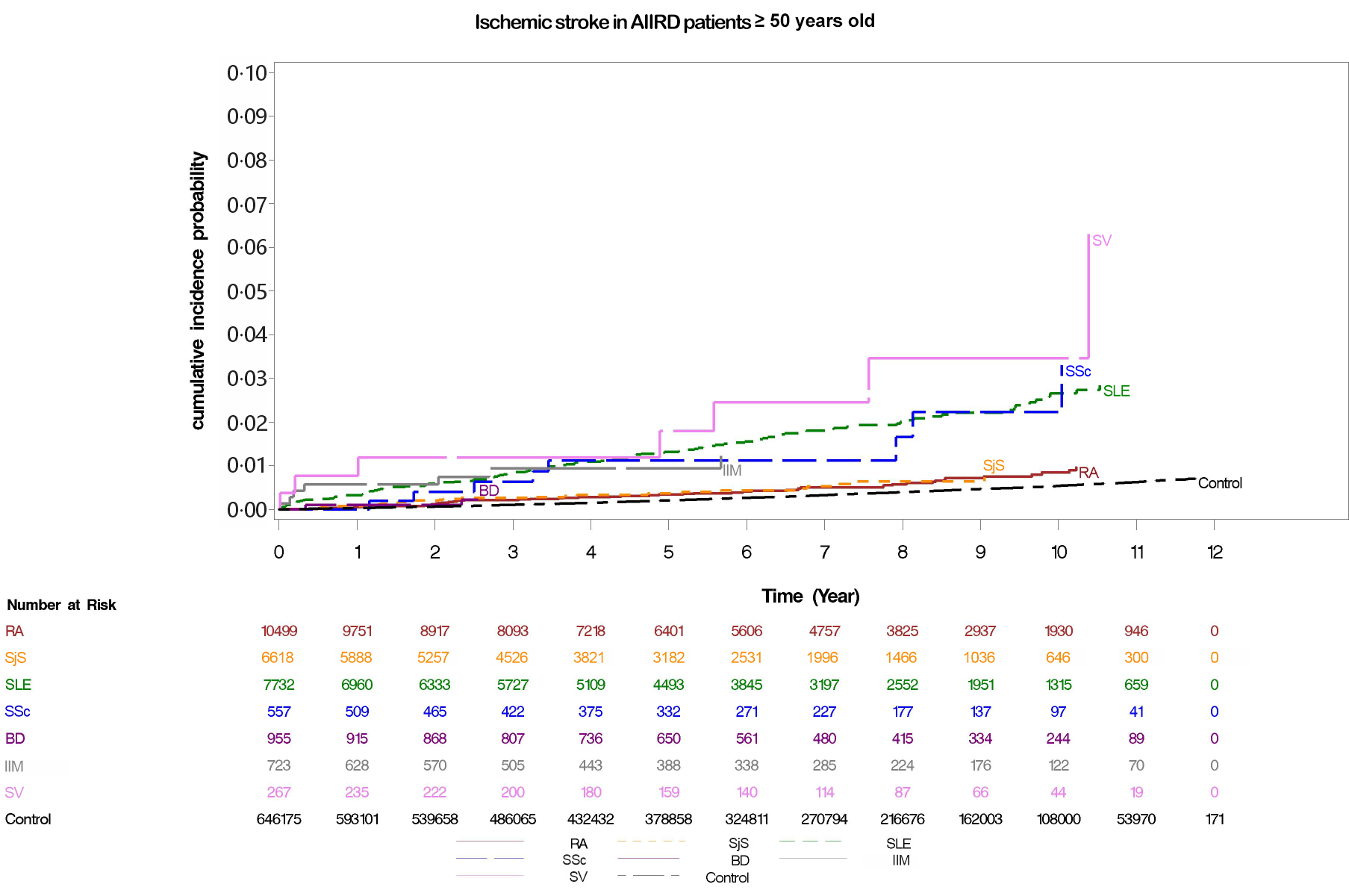

Supplement: Supplementary Figure 1 — Cumulative incidences of ischemic stroke in patients with autoimmune inflammatory rheumatic diseases (AIIRDs) ≥50 years old, estimated via the Kaplan–Meier method. RA, rheumatoid arthritis; SjS, Sjögren’s syndrome; SLE, systemic lupus erythematosus; IIM, idiopathic inflammatory myositis; SSc, systemic sclerosis; BD, Behçet’s disease; SV, systemic vasculitis. [file Image_1.tif]
